# Hypoxia Potentiates the Radiation-Sensitizing Effect of Olaparib in Human Non-Small Cell Lung Cancer Xenografts by Contextual Synthetic Lethality

**DOI:** 10.1016/j.ijrobp.2016.01.035

**Published:** 2016-06-01

**Authors:** Yanyan Jiang, Tom Verbiest, Aoife M. Devery, Sivan M. Bokobza, Anika M. Weber, Katarzyna B. Leszczynska, Ester M. Hammond, Anderson J. Ryan

**Affiliations:** Cancer Research UK/Medical Research Council Oxford Institute for Radiation Oncology, Department of Oncology, University of Oxford, Oxford, United Kingdom

## Abstract

**Purpose:**

Poly(ADP-ribose) polymerase (PARP) inhibitors potentiate radiation therapy in preclinical models of human non-small cell lung cancer (NSCLC) and other types of cancer. However, the mechanisms underlying radiosensitization in vivo are incompletely understood. Herein, we investigated the impact of hypoxia on radiosensitization by the PARP inhibitor olaparib in human NSCLC xenograft models.

**Methods and Materials:**

NSCLC Calu-6 and Calu-3 cells were irradiated in the presence of olaparib or vehicle under normoxic (21% O_2_) or hypoxic (1% O_2_) conditions. In vitro radiosensitivity was assessed by clonogenic survival assay and γH2AX foci assay. Established Calu-6 and Calu-3 subcutaneous xenografts were treated with olaparib (50 mg/kg, daily for 3 days), radiation (10 Gy), or both. Tumors (n=3/group) were collected 24 or 72 hours after the first treatment. Immunohistochemistry was performed to assess hypoxia (carbonic anhydrase IX [CA9]), vessels (CD31), DNA double strand breaks (DSB) (γH2AX), and apoptosis (cleaved caspase 3 [CC3]). The remaining xenografts (n=6/group) were monitored for tumor growth.

**Results:**

In vitro, olaparib showed a greater radiation-sensitizing effect in Calu-3 and Calu-6 cells in hypoxic conditions (1% O_2_). In vivo, Calu-3 tumors were well-oxygenated, whereas Calu-6 tumors had extensive regions of hypoxia associated with down-regulation of the homologous recombination protein RAD51. Olaparib treatment increased unrepaired DNA DSB (*P*<.001) and apoptosis (*P*<.001) in hypoxic cells of Calu-6 tumors following radiation, whereas it had no significant effect on radiation-induced DNA damage response in nonhypoxic cells of Calu-6 tumors or in the tumor cells of well-oxygenated Calu-3 tumors. Consequently, olaparib significantly increased radiation-induced growth inhibition in Calu-6 tumors (*P*<.001) but not in Calu-3 tumors.

**Conclusions:**

Our data suggest that hypoxia potentiates the radiation-sensitizing effects of olaparib by contextual synthetic killing, and that tumor hypoxia may be a potential biomarker for selecting patients who may get the greatest benefit from the addition of olaparib to radiation therapy.

**Summary**

We show that the PARP inhibitor olaparib enhances radiation-induced DNA damage response in hypoxic tumor cells of Calu-6 xenografts but not in nonhypoxic tumor cells of Calu-6 xenografts or in tumor cells of well-oxygenated Calu-3 xenografts. Consequently, olaparib potentiates the antitumor effect of radiation in hypoxic tumors but not in well-oxygenated tumors. This suggests that hypoxia enhances the radiation-sensitizing effects of olaparib in human non-small cell lung cancer (NSCLC) xenografts by contextual synthetic lethality.

## Introduction

Non-small cell lung cancer (NSCLC), accounting for 80% to 85% of lung cancer cases, is the leading cause of cancer-related death worldwide (1). A third of NSCLC patients are diagnosed at a locally advanced stage and radiation therapy is a mainstay for the treatment of these patients 2, 3. Despite technical advances in radiation therapy, the local tumor control remains suboptimal due to radioresistance 4, 5. Because repair of DNA damage is a major cause of radioresistance, inhibition of DNA repair represents a rational strategy to enhance the efficacy of radiation therapy.

Poly(ADP-ribose) polymerase 1 (PARP-1) is a DNA damage–sensing protein. PARP-1 is activated upon binding to DNA single-strand breaks (SSBs), whereupon it catalyzes the production of poly(ADP-ribose) chains attached to itself and other nearby proteins in order to facilitate DNA repair (6). Thus this enzyme has been considered a promising target for radiosensitization. Indeed, PARP inhibition has been shown to enhance the effects of radiation therapy in various cell lines and several pre-clinical tumor types including colon cancer 7, 8, prostate cancer (9), breast cancer (10), and NSCLC 9, 10, 11. The mechanism for radiosensitization by PARP inhibition in vitro is well understood. PARP inhibitors prevent the release of PARP from radiation-damaged DNA, thus DNA repair proteins cannot access the SSBs. Unrepaired SSBs are converted to lethal DNA double-strand breaks (DSBs) during replication, thereby enhancing radiotoxicity (12).

Nevertheless, the underlying mechanisms of radiosensitization by PARP inhibition in vivo remain incompletely understood. To date, few studies have looked at the role of tumor microenvironment in radiosensitization by PARP inhibition, although effects on the vasculature, such as decreases in tumor vascular density (13) or tumor vascular perfusion (11), have been implicated in potentiation of radiation therapy by PARP inhibition in lung cancer xenografts.

Tumor hypoxia is a major microenvironmental factor that limits radiation therapy efficacy by decreasing oxygen-mediated free radical damage (14). Chronic hypoxia suppresses homologous recombination (HR) protein expression and function in tumor cells 15, 16. As HR is required for repair of DSBs that result from PARP inhibition 17, 18, impaired HR can lead to unrepaired DSBs and subsequently cell death. Therefore, hypoxia-induced HR defects are considered synthetically lethal with PARP inhibition 19, 20. This microenvironment-mediated synthetic killing has been termed “contextual synthetic lethality” (19). Such contextual synthetic lethality might be synergistic with radiation. Despite several in vitro studies showing that PARP inhibition radiosensitizes cells under chronic hypoxia conditions 9, 20, 21, hypoxia has not yet been directly linked to the radiosensitization by PARP inhibition in solid tumors in vivo.

In this study, we sought to investigate the impact of hypoxia on the radiosensitization by PARP inhibition in human NSCLC xenograft tumors grown in nude mice. We assessed the DNA damage response and tumor growth inhibition in 2 NSCLC xenograft models: Calu-6 xenografts (hypoxic) and Calu-3 xenografts (well-oxygenated), following radiation and the PARP1/2 inhibitor olaparib treatment.

## Methods and Materials

### Cell culture

Human lung carcinoma cells Calu-3, Calu-6, and A549 were purchased from the American Type Culture Collection. Cells were cultured in advanced Dulbecco's modified Eagle medium/F12 medium supplemented with 5% fetal bovine serum, 2 mM glutamax, and 50 μg/mL penicillin/streptomycin in a humidified atmosphere with 7.5% CO_2_.

### In vitro γH2AX foci assay

The γH2AX foci assay was performed as previously described (22). Briefly, cells were seeded into 96-well plates and treated with 5 μmol/L olaparib or 0.05% dimethyl sulfoxide (DMSO) 1 hour before irradiation (0 or 10 Gy) and then returned to the incubator for 24 hours before fixation. Cells were fixed with 3% paraformaldehyde, and blocked with 1% goat serum/1% bovine serum albumin/0.1% Triton X-100 followed by incubation with γH2AX antibody (1:1500 dilution; Millipore). Cells were then washed and incubated with Alexa 488-labeled goat anti-mouse antibody (1:1200 dilution; Invitrogen) and Hoechst (5 μg/mL; Sigma-Aldrich). The number of γH2AX foci per nucleus was quantified from 400 nuclei per well using the In Cell analyzer 1000 (GE Healthcare).

### Clonogenic survival assays

Exponentially growing cells were seeded into 6-well plates, and exposed to 21% O_2_ (normoxia) or 1% O_2_ (hypoxia) in a Whitley H35 Hypoxystation (Don Whitley Scientific Ltd) for 16 hours. An amount of 5 μmol/L olaparib (Selleck) or 0.05% DMSO was added to the medium 1 hour before irradiation (0-6 Gy) with a cesium-137 source at 1.69 Gy/min (GSR D1 irradiator; Gamma Service Ltd) under normoxic or hypoxic conditions. Twenty-four hours after irradiation, cells were washed, and fresh medium was added. Plates were incubated for 6 to 13 days to allow colonies to form. Colonies were then fixed and stained with 0.5% crystal violet in 5% acetic acid and 75% methanol. Colonies (≥50 cells) were counted. Clonogenic survival curves and the sensitization enhancement ratio at 50% (SER_50_) were generated using OriginPro version 8.5.1 software (OriginLab Corp).

### Subcutaneous xenografts

All animal experiments were performed following local ethical review under a project license from the UK Home Office. Subject animals, 6- to 8-week-old female BALB/c nude mice (Harlan Labs), were anesthetized with 2% isoflurane. Calu-6 cells (2 × 10^6^), Calu-3 cells (5 × 10^6^), or A549 cells (2 × 10^6^) in 50% Matrigel (BD Biosciences) were subcutaneously injected into a single site on the back of the mouse. Mouse weights and tumor volumes were measured 3 times each week (volume = 1/2 × length × width × depth).

### Tumor irradiation and treatment schedules

When xenografts reached 100 mm^3^, mice were randomized to 1 of 4 groups (n=12/group): (*1*) the vehicle group (10% DMSO/10% captisol/PBS daily for 3 days by intraperitoneal injection); (*2*) the olaparib group (olaparib 50 mg/kg daily for 3 days by intraperitoneal injection); (*3*) the irradiation group (tumor-localized irradiation at a single dose of 10 Gy [RS320 irradiation system; 1.82 Gy/min; Gulmay Medical Ltd) as previously described (23); and (*4*) the combination group (olaparib 50 mg/kg daily for 3 days, and tumor irradiation [10 Gy] given 30 minutes after the first dose of olaparib).

Three animals from each group were sacrificed at 24 or 72 hours after the first treatment, respectively, and tumors were processed for immunohistochemistry (IHC). The remaining animals (n=6/group) were monitored for tumor growth until the sizes of tumors reached 500 mm^3^ or 42 days after initiation of treatment, whichever occurred first.

### Immunohistochemistry assay

Paraformaldehyde-fixed and paraffin-embedded tumor sections were stained using EnVision G2 doublestain system (Dako) according to the manufacturer's instructions. Antibodies used for immunohistochemistry were carbonic anhydrase IX (CA9; 1:800 dilution; product AB1001; BioScience), CD31 (1:50 dilution; product ab28364; Abcam), GLUT1 (1:200 dilution; product ab14683; Abcam), RAD51 (1:200 dilution; product GTX70230; GeneTex Inc), γH2AX (1:1000 dilution; product 05-636; Millipore), and cleaved caspase 3 (CC3; 1:600 dilution; product 9661; Cell Signaling Technology). Staining for CC3, γH2AX, RAD51 (3,3′-diaminobenzidine) and CA9 (Permanent Red) in whole-tumor sections (excluding necrotic areas) was analyzed using an Aperio CS scanner and ImageScope analysis software (Aperio Technologies). Three tumor samples were used per group. For γH2AX foci quantification, 200 nuclei were assessed from each tumor sample. EF5 binding was detected using the Cy3-conjugated antibody ELK3-51 (75 mM; University of Pennsylvania) as described previously (24).

### Statistical analysis

Data were expressed as means ± SEM. Statistical analysis was performed using Student *t* test (2-groups comparison) or one-way ANOVA (more than 2 groups comparison) by SPSS software (IBM). Statistical significance was defined as *P*<.05.

## Results

### Olaparib enhances cytotoxic effects of radiation in human NSCLC cells in vitro

The radiation-sensitizing effect of olaparib on normoxic NSCLC cell lines in vitro was reported previously (11). To assess whether olaparib affected DNA repair following radiation, residual DNA DSBs in Calu-6 and Calu-3 cells were quantified using a γH2AX foci immunofluorescence assay (25) 24 hours after irradiation in the presence of olaparib or vehicle (Fig. E1; available online at www.redjournal.org). As expected, radiation alone markedly increased the residual DNA DSBs in both cell lines. Olaparib alone also led to an increase in residual γH2AX foci formation (*P*<.01). The combination treatment significantly increased residual DNA DSBs compared with radiation alone (*P*<.01), suggesting that olaparib compromises the repair of radiation-induced DNA damage.

**Fig. 1:**
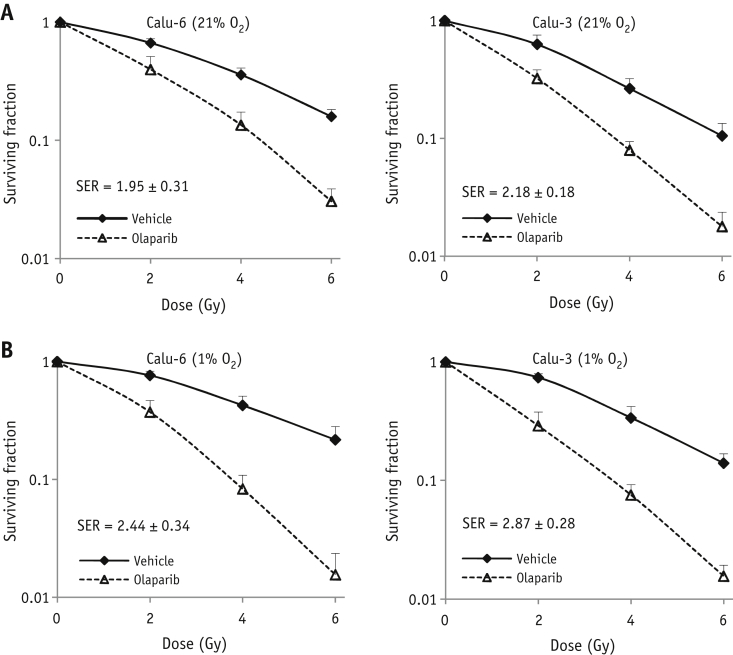
Olaparib radiosensitizes NSCLC cell lines in vitro. Calu-6 and Calu-3 cells were exposed to normoxia (21% O_2_) or hypoxia (1% O_2_). Olaparib or vehicle was added to the medium 1 hour before radiation. At 24 hours after radiation, cells were cultured in drug-free medium for clonogenic survival assays. (A) Clonogenic survival curves under normoxia. (B) Clonogenic survival curves under hypoxia. *Abbreviation:* SER = sensitization enhancement ratio.

To further investigate the impact of oxygen on the radiation-sensitizing potential of olaparib in NSCLC cell lines, clonogenic survival assays were performed on irradiated Calu-6 and Calu-3 cells in the presence of olaparib or vehicle under 21% O_2_ (normoxic) or 1% O_2_ (hypoxic) conditions. We observed a dose-dependent decrease of the surviving fraction in both normoxic and hypoxic cell lines. Although chronic hypoxic Calu-3 and Calu-6 cells were slightly more radioresistant than normoxic cells, the radiation-sensitizing effect of olaparib is greater under chronic hypoxia (SER: 2.87 ± 0.28 for Calu-3 and SER: 2.44 ± 0.34 for Calu-6) than under normoxia (SER: 2.18 ± 0.18 for Calu-3 and SER: 1.95 ± 0.31 for Calu-6) (Fig. 1A, B), suggesting that hypoxia induces contextual synthetic lethality with PARP inhibition in vitro.

### Calu-6 xenografts have extensive hypoxic regions

To investigate the effects of hypoxia on radiosensitization by olaparib in vivo, subcutaneous Calu-6 and Calu-3 xenografts were established, and tumor hypoxia was assessed by IHC staining for CA9, a widely used intrinsic marker for tumor hypoxia (26). The utility of CA9 as a hypoxia marker was confirmed using another intrinsic hypoxia marker GLUT1 and the extrinsic hypoxia marker EF5 in sequential Calu-6 tumor sections. Good spatial correlation was observed among CA9, GLUT1, and EF5 staining in Calu-6 xenograft tumors (Fig. E2; available online at www.redjournal.org).

**Fig. 2:**
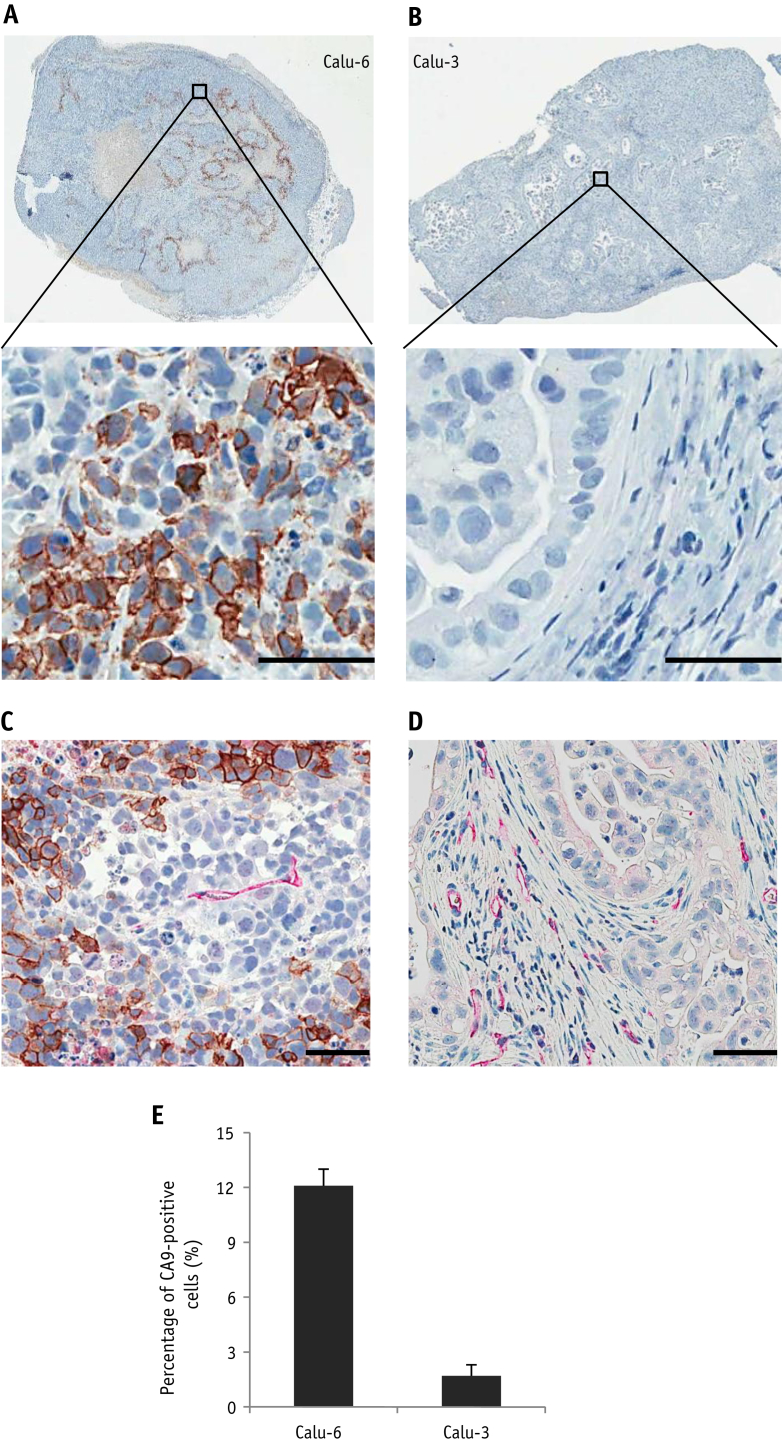
Hypoxia and vasculature in NSCLC tumors. (A) Representative CA9 staining in Calu-6 tumors. (B) Representative CA9 staining in Calu-3 tumors. (C) Representative CA9 (brown)/CD31 (red) co-staining in Calu-6 tumors. (D) Representative CA9 (brown)/CD31 (red) co-staining in Calu-3 tumors. (E) Quantitative analysis of the percentage of hypoxic cells in Calu-6 and Calu-3 tumors. *Abbreviations:* Bars = 50 μm; n=3/group. (A color version of this figure is available at www.redjournal.org.)

Calu-6 tumors showed extensive plasma membrane staining for CA9 in contiguous tumor cells bordering regions of necrosis (Fig. 2A), with 12.1% ± 0.9% of cells in Calu-6 tumors positively stained with CA9 (Fig. 2E), demonstrating that Calu-6 tumors contain significant areas of hypoxia. In contrast, CA9 staining was rarely seen in Calu-3 xenograft tumor cells (Fig. 2B), with staining evident only in occasional isolated cells. 1.7% ± 0.6% of tumor cells in Calu-3 tumors were positively stained with CA9 (Fig. 2E), indicating that Calu-3 xenograft tumors are well-oxygenated. We have previously shown that Calu-6 xenografts are more poorly perfused than Calu-3 xenografts (23). Consistent with this, the blood vessels in Calu-6 xenografts are sparse and embedded within tumor cell mass surrounded by regions of hypoxia (Fig. 2C), whereas there is a high density of blood vessels within the stroma of nonhypoxic Calu-3 xenografts (Fig. 2D).

### RAD51 expression is down-regulated in hypoxic regions of Calu-6 xenografts

**Fig. 3:**
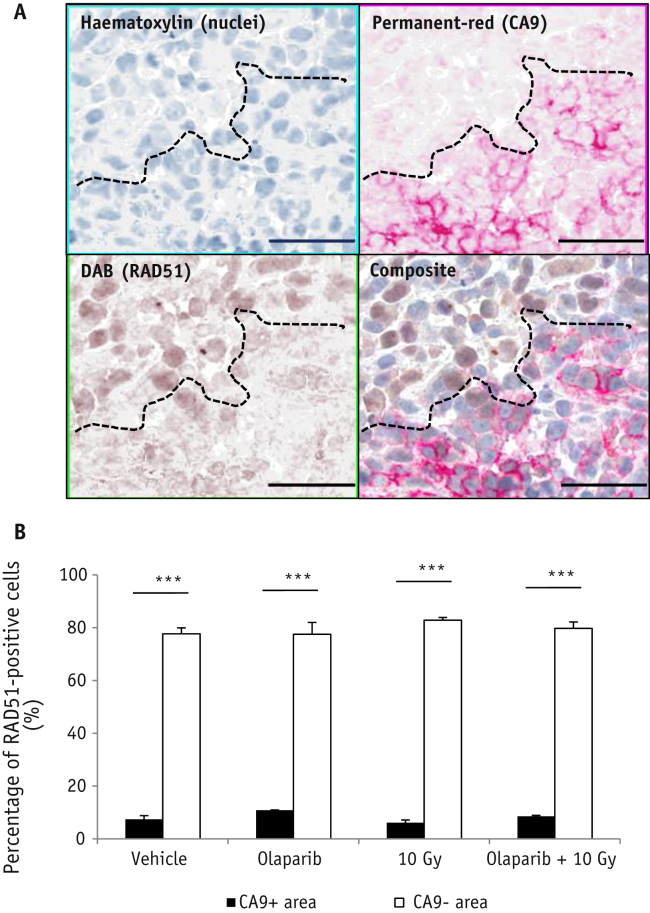
RAD51 protein expression is reduced in hypoxic tumor cells. Mice bearing Calu-6 xenografts were treated with olaparib (50 mg/kg intraperitoneal) or vehicle 30 minutes prior to radiation (0 or 10 Gy). Tumors were collected 24 hours post-radiation for IHC. (A) Representative RAD51 (brown)/CA9 (red) staining in a vehicle-treated Calu-6 tumor. Bar = 50 μm. (B) Percentage of RAD51-positive cells in CA9-positive (hypoxic) and CA9-negative (nonhypoxic) tumor regions (mean ± SEM). ***P<.001. n=3/group. *Abbreviation:* IHC = immunohistochemistry. (A color version of this figure is available at www.redjournal.org.)

Hypoxia can decrease HR protein expression. To explore whether hypoxia-induced down-regulation of HR protein expression was apparent in Calu-6 tumors, tumors excised 24 hours post-treatment were stained for RAD51 and CA9 (Fig. 3A). The percentage of RAD51-positive cells was significantly lower in CA9-positive areas (hypoxic) compared with CA9-negative areas (nonhypoxic) (*P*<.001), irrespective of treatment (Fig. 3B).

### Olaparib treatment increases unrepaired DNA DSBs and apoptosis in hypoxic tumor cells of Calu-6 xenografts following irradiation

**Fig. 4:**
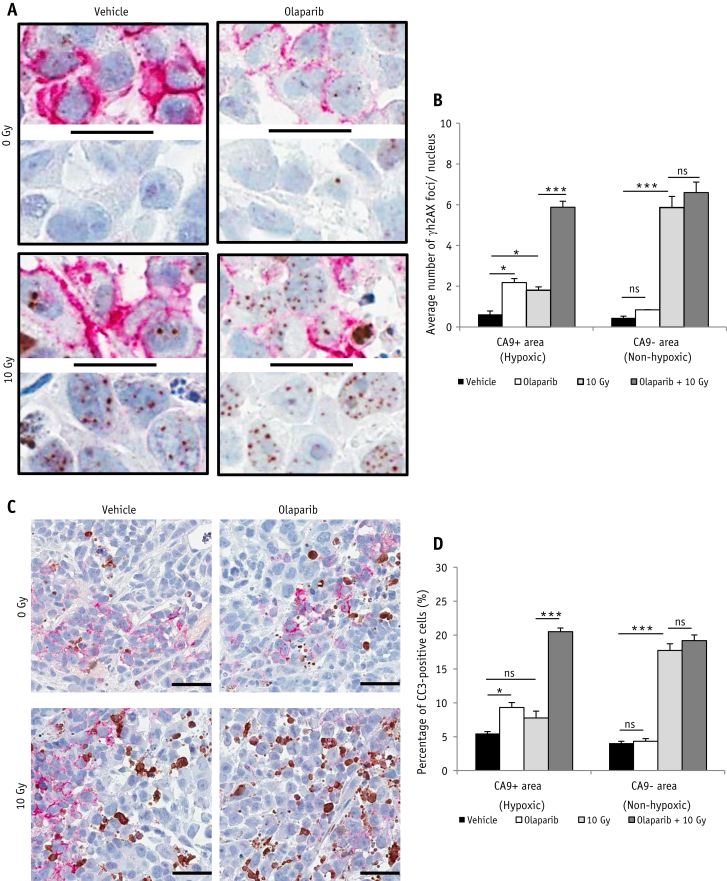
Olaparib increases unrepaired DNA DSBs and apoptosis in hypoxic tumor cells of Calu-6 xenografts following radiation. Mice bearing Calu-6 xenografts were treated with olaparib (50 mg/kg) or vehicle 30 min prior to 0- or 10-Gy radiation. Tumors were collected 24 or 72 hours post-radiation for IHC. (A) Representative γH2AX (brown)/CA9 (red) staining at 24 hours. Bar = 20 μm. (B) Quantitative analysis of the γH2AX foci number per nucleus in CA9-positive and -negative tumor subregions (mean ± SEM). (C) Representative CC3 (brown)/CA9 (red) staining at 72 hours. Bar = 50 μm. (D) Quantitative analysis of the percentage of CC3-positive cells in CA9-positive and -negative tumor subregions (mean ± SEM). *P<.05, ***P<.001. n=3/group. *Abbreviations:* DSB = double-strand breaks; IHC = immunohistochemistry; ns = not significant. (A color version of this figure is available at www.redjournal.org.)

We next addressed the question of whether olaparib exerted differential effects on radiation response in hypoxic and nonhypoxic Calu-6 cells in vivo. γH2AX foci levels were used as a surrogate of residual (unrepaired) DNA DSBs (27) and assessed by IHC 24 hours post-treatment (Fig. 4A). As expected, radiation alone induced a significant increase of γH2AX foci in CA9-negative (nonhypoxic) cells (*P*<.001), but induced much fewer foci in CA9-positive (hypoxic) cells (Fig. 4B). Olaparib alone led to elevated γH2AX foci levels in CA9-positive (hypoxic) cells (*P*<.05), but not in CA9-negative cells (Fig. 4B). Surprisingly, when combined with radiation, γH2AX foci were markedly increased in CA9-positive (*P*<.001), but not in CA9-negative cells compared with radiation alone. These data suggest that olaparib selectively impacts the radiation-induced DNA damage response in hypoxic cancer cells. We also extended these data into another hypoxic NSCLC xenograft model (A549) and showed that olaparib selectively increased residual γH2AX foci in CA9-positive tumor cells 24 hours after radiation (Fig. E3; available online at www.redjournal.org).

Calu-6 tumors were stained for CC3, a protease involved in programmed cell death and a cellular marker of apoptosis (28) (Fig. 4C). Consistent with the γH2AX foci data, radiation alone significantly increased the apoptosis rate in CA9-negative cells (*P*<.001), but not in CA9-positive cells. Olaparib alone increased apoptosis rate in CA9-positive cells (*P*<.05), and when in combination with radiation, apoptosis was significantly enhanced in CA9-positive cells (*P*<.001), but not in CA9-negative cancer cells, compared with radiation alone (Fig. 4D). These data suggest that olaparib enhances radiation-induced cell killing through induction of apoptosis in hypoxic cancer cells.

### Olaparib does not enhance DNA damage response to radiation in Calu-3 xenografts

Effects of olaparib on DNA damage response to radiation were also assessed in Calu-3 xenografts. Since CA9 was hardly detected in Calu-3 tumors, γH2AX foci and apoptotic cells were only quantified in CA9-negative cells. Twenty-four hours after treatment, olaparib showed no effect on levels of γH2AX foci compared with vehicle-treated controls, and there was no significant difference in the numbers of γH2AX foci and CC3-positive cells between the radiation treatment group and the combination treatment group (Fig. E4; available online at www.redjournal.org). This finding, together with the data in Calu-6 xenografts above suggests that olaparib does not significantly affect the DNA damage response to radiation in nonhypoxic cancer cells in vivo.

### Olaparib treatment increases radiation-induced growth inhibition in Calu-6 but not in Calu-3 xenografts

**Fig. 5:**
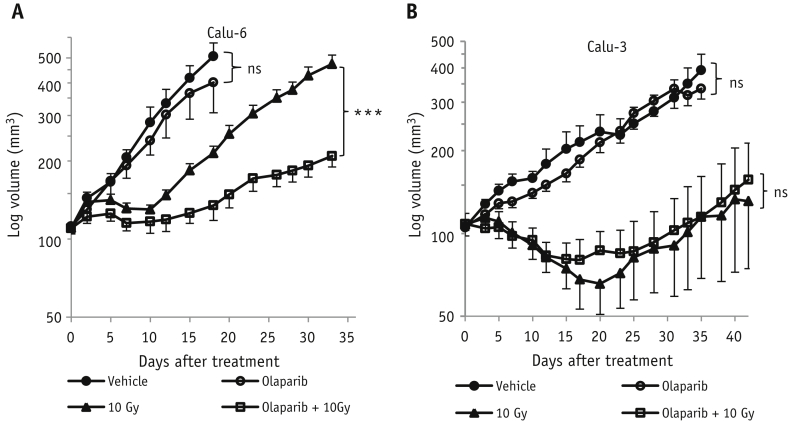
Combination of radiation with olaparib induces significant growth inhibition in Calu-6 xenografts but not in Calu-3 xenografts. Tumor-bearing mice were randomized (n=6/group) to receive vehicle, olaparib (50 mg/kg), 10-Gy radiation, or olaparib combined with radiation. The growth curves of Calu-6 xenografts (A) and Calu-3 xenografts (B) were plotted (mean ± SEM). ***P<.001. n=3/group. *Abbreviation:* ns = not significant.

Given the finding that olaparib potentiated radiation-induced DNA damage response in hypoxic Calu-6 tumor cells in vivo, we next examined how olaparib affected the antitumor effect of radiation in NSCLC tumors. Compared to Calu-3 tumors, Calu-6 tumors are more aggressive, possibly associated with sustained tumor hypoxia. For Calu-6 xenografts, olaparib alone had a small but nonsignificant effect on tumor growth compared with vehicle-treated controls (*P*=.35, Fig. 5A). In combination with radiation, tumor growth inhibition was significantly enhanced compared with radiation alone (*P*<.001, Fig. 5A). In Calu-3 xenografts, olaparib alone had no evident effect on the tumor growth (*P*=.39), and combination treatment did not produce any greater antitumor effect than radiation alone (*P*=.53) (Fig. 5B). Therefore, olaparib was not effective as a monotherapy, even in Calu-6 tumors that have a significant proportion of hypoxic cells. However, our results suggest that olaparib can act by selectively increasing the antitumor effect of radiation in hypoxic regions of tumors.

## Discussion

Although PARP inhibition has shown radiation-sensitizing effects, the underlying mechanism in the context of hypoxia in vivo has not yet been reported. Here, we show that in vitro, radiation-sensitizing effect of olaparib was greater under hypoxia than normoxia in both Calu-3 and Calu-6 NSCLC cell lines. In vivo, olaparib increases radiation-induced residual DNA DSBs and apoptosis in the hypoxic tumor cells of Calu-6 xenografts, but not in the nonhypoxic tumor cells of Calu-6 xenografts or in the tumor cells of well-oxygenated Calu-3 xenografts. As a result, olaparib potentiates antitumor effects of radiation in Calu-6 xenografts but not in Calu-3 xenografts. These findings suggest that the radiosensitization by olaparib in vivo may be due to hypoxia-induced contextual synthetic killing.

Synergistic effects of PARP inhibitors in combination with radiation therapy have been reported in human NSCLC xenografts, including Calu-6, A549, and H460 xenografts 10, 11, 13. According to our own findings and studies carried out by other groups 29, 30, these xenograft models reported contain substantial regions of hypoxia, whereas no previous work has investigated the effect of the combination treatment in nonhypoxic NSCLC tumor models in vivo. Barreto-Andrade et al (31) have noted that PARP inhibition by ABT-888 enhances antitumor effects of radiation in PC-3 prostate tumors but not in DU-145 prostate tumors, despite similar radiation sensitization shown in both cell lines in vitro. Another study has shown that the average hypoxic fraction of PC-3 xenografts (52%) is higher than that of DU-145 xenografts (7%) (32), but this aspect was not investigated by Barreto-Andrade et al (31) in their study. Taken together, the published literature supports our finding that the sensitizing effect of olaparib on radiation is largely restricted to hypoxic regions of tumors.

RAD51 plays a central role in HR. In the present study, we observed downregulation of RAD51 in hypoxic Calu-6 tumor cells in vivo. Our results are consistent with other reports that have shown chronic hypoxia reduces HR protein expression, leading to functional impairment of the HR pathway of DNA DSB repair 15, 16, 33. Moreover, it has been shown that radiosensitization by PARP inhibition is more effective in DNA DSB repair-defective tumor cells (34). Therefore, hypoxia-induced “contextual synthetic lethality” can be synergistic with radiation.

Our observation that olaparib exhibited greater radiation-sensitizing effect under hypoxia than under normoxia in vitro is in agreement with others' finding (35). However, no evidence of radiosensitization was observed for nonhypoxic tumor cells in vivo. This discrepancy may be due to the tumor microenvironmental factors that could change the sensitivity of cells to olaparib combined with radiation. In addition, in vitro data from other groups suggest that the phase of the cell cycle affects PARP inhibition–induced radiosensitization. Cells in S/G_2_ phases show greatest sensitivity to PARP inhibition combined with radiation, whereas noncycling cells exhibit minimal sensitivity 36, 37. It is possible that local tumor microenvironment changed the proportion of the proliferating versus nonproliferating tumor cells compared with tumor cells grown in vitro, leading to a change in the radiation-sensitizing effect. However, the underlying mechanism may be more complex and will require further studies.

Chan et al (19) previously showed that PARP inhibition alone selectively killed hypoxic mouse embryonic fibroblasts and thus proposed that PARP inhibitors might be used as a monotherapy for hypoxic tumors. However, in our study, although olaparib alone moderately enhanced DNA damage response in hypoxic Calu-6 tumor cells, it did not significantly inhibit tumor growth, suggesting that the hypoxic fraction of the tumor was insufficient to achieve a significant antitumor effect by olaparib monotherapy.

Hypoxia contributes to aggressive tumor growth 38, 39, and hypoxic cells are resistant to radiation (40). Our data suggest that treatment-resistant hypoxic cells can be targeted by using PARP inhibition in combination with radiation therapy. Moreover, tumor hypoxia might be predictive for antitumor effects of the combination treatment. This will be particularly useful in lung cancer treatment, as the oxygenation in lung cancer varies 41, 42 and 25% of NSCLC cases can be defined as highly hypoxic (43). Interestingly, a higher level of hypoxia was found in squamous carcinoma, indicating this subtype of NSCLC might respond well to the combination treatment.

It is interesting to note that the levels of residual γH2AX foci and apoptosis in Calu-3 tumors were lower than Calu-6 tumors following radiation. We and others have previously shown that Calu-3 and Calu-6 xenograft models display different stromal phenotypes 23, 44, which might result in differential DNA damage response to radiation in Calu-3 and Calu-6 xenografts.

In this study, we used CA9 immunostaining as a marker for hypoxia, which correlated with EF5 staining. Noninvasive in vivo imaging of hypoxia using hypoxic specific PET radiotracers, such as [^18^F]FMISO, [^18^F]FAZA, and [^18^F]EF5 (45), are potential modalities for patient selection. In addition, a recently developed in vivo hypoxia metagene signature might provide another method to identify hypoxic tumors 46, 47.

## Conclusions

In conclusion, our study suggests that hypoxia potentiates radiation-sensitizing effects of PARP inhibitor olaparib by contextual synthetic lethality. Tumor hypoxia may be a potential predictive biomarker that could identify the subset of patients who would benefit most from a combination treatment of olaparib and radiation therapy.

## Supplementary Data

Figure E1

Figure E2

Figure E3

Figure E4
