# Supplementary material for: Hypoxia Potentiates the Radiation-Sensitizing Effect of Olaparib in Human Non-Small Cell Lung Cancer Xenografts by Contextual Synthetic Lethality
Source: Int J Radiat Oncol Biol Phys. 2016 Jun 1;95(2):772–81. doi: 10.1016/j.ijrobp.2016.01.035 (PMC4856738; doi:10.1016/j.ijrobp.2016.01.035)
Supplement: Figure E1 [file mmc1.pdf]

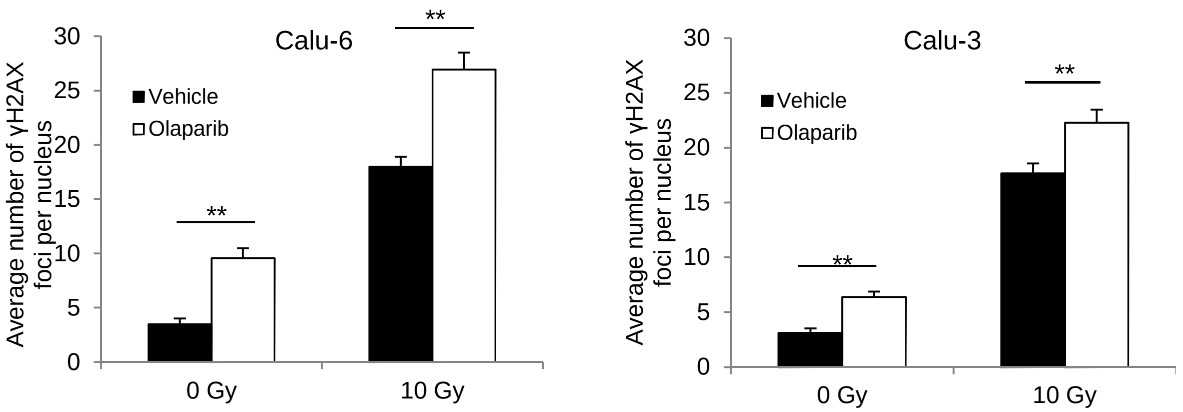

Supplementary Fig. S1 immunofluorescence staining of γH2AX foci in NSCLC cell lines. Calu-6 and Calu-3 cells were incubated with olaparib or vehicle starting 1 h before radiation (0 or 10 Gy). Cells were fixed 24 h post-radiation for γH2AX immunofluorescence staining. The average numbers of γH2AX foci per nucleus were quantified. Data were represented as means ± SEM from three independent experiments. $^{**}P<0.01$.

Supplementary Fig. S1
