# Supplementary material for: Hypoxia Potentiates the Radiation-Sensitizing Effect of Olaparib in Human Non-Small Cell Lung Cancer Xenografts by Contextual Synthetic Lethality
Source: Int J Radiat Oncol Biol Phys. 2016 Jun 1;95(2):772–81. doi: 10.1016/j.ijrobp.2016.01.035 (PMC4856738; doi:10.1016/j.ijrobp.2016.01.035)
Supplement: Figure E2 [file mmc2.pdf]

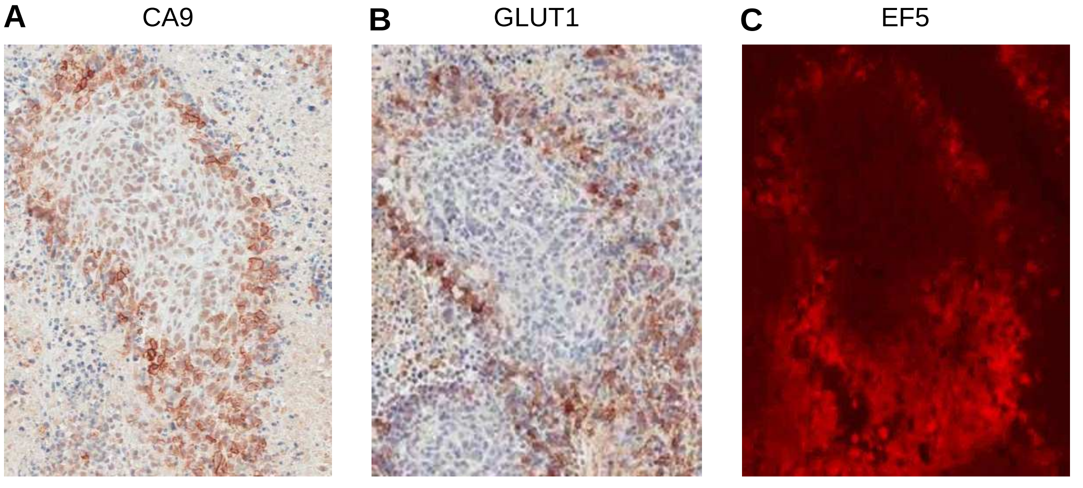

Supplementary Fig. S2 Immunohistological detection of tumor hypoxia. Mice bearing Calu-6 xenografts were intraperitoneally injected with EF5 2 h prior to sacrifice. Tumors were then collected and cut into serial sections for IHC. (A) Stained with anti-CA9 antibody (brown). (B) Stained with anti-GLUT1 antibody (brown). (C) Stained with Cy3-labeled anti-EF5 antibody (red).

Supplementary Fig. S2
