# Supplementary material for: Hypoxia Potentiates the Radiation-Sensitizing Effect of Olaparib in Human Non-Small Cell Lung Cancer Xenografts by Contextual Synthetic Lethality
Source: Int J Radiat Oncol Biol Phys. 2016 Jun 1;95(2):772–81. doi: 10.1016/j.ijrobp.2016.01.035 (PMC4856738; doi:10.1016/j.ijrobp.2016.01.035)
Supplement: Figure E3 [file mmc3.pdf]

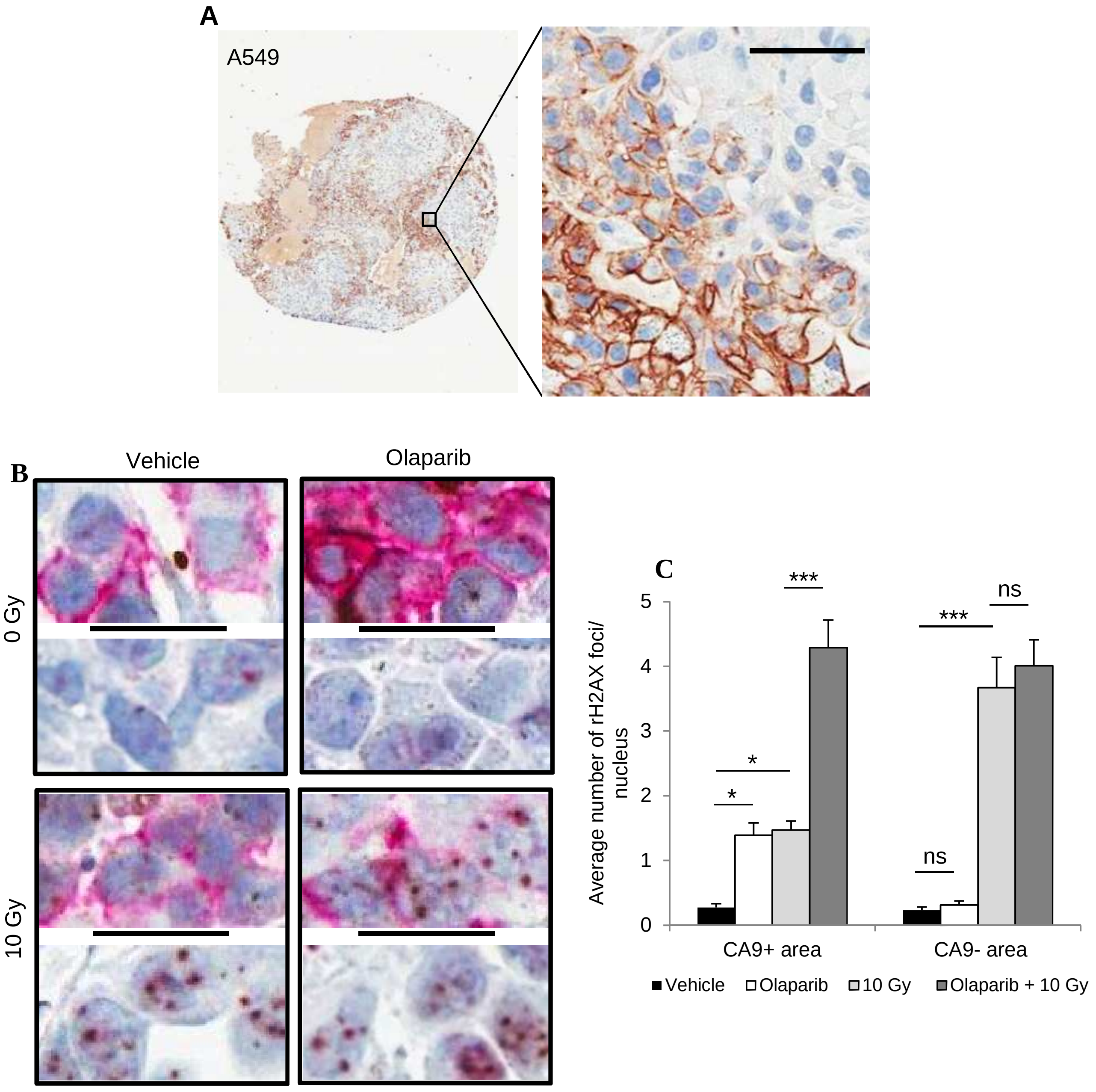

Supplementary Fig. S3. Olaparib increases residual DNA DSBs in hypoxic tumor cells of A549 xenografts following radiation. Mice bearing established A549 xenografts (100 mm^3) were treated with olaparib or vehicle 30 min prior to 0 Gy or 10 Gy radiation. Tumors were collected 24 h post-radiation for CA9 and γH2AX IHC staining. (A) Representative CA9 staining in A549 tumors. Scale bar, 50 μm. (B) Representative γH2AX (brown)/ CA9 (red) staining. Scale bars, 20 μm. (C) Quantitative analysis of the average γH2AX foci per nucleus in CA9 positive and negative tumor subregions (mean ± SEM). ns: not significant, *P<0.05, ***P<0.001.

Supplementary Fig. S3
