# Supplementary material for: Hypoxia Potentiates the Radiation-Sensitizing Effect of Olaparib in Human Non-Small Cell Lung Cancer Xenografts by Contextual Synthetic Lethality
Source: Int J Radiat Oncol Biol Phys. 2016 Jun 1;95(2):772–81. doi: 10.1016/j.ijrobp.2016.01.035 (PMC4856738; doi:10.1016/j.ijrobp.2016.01.035)
Supplement: Figure E4 [file mmc4.pdf]

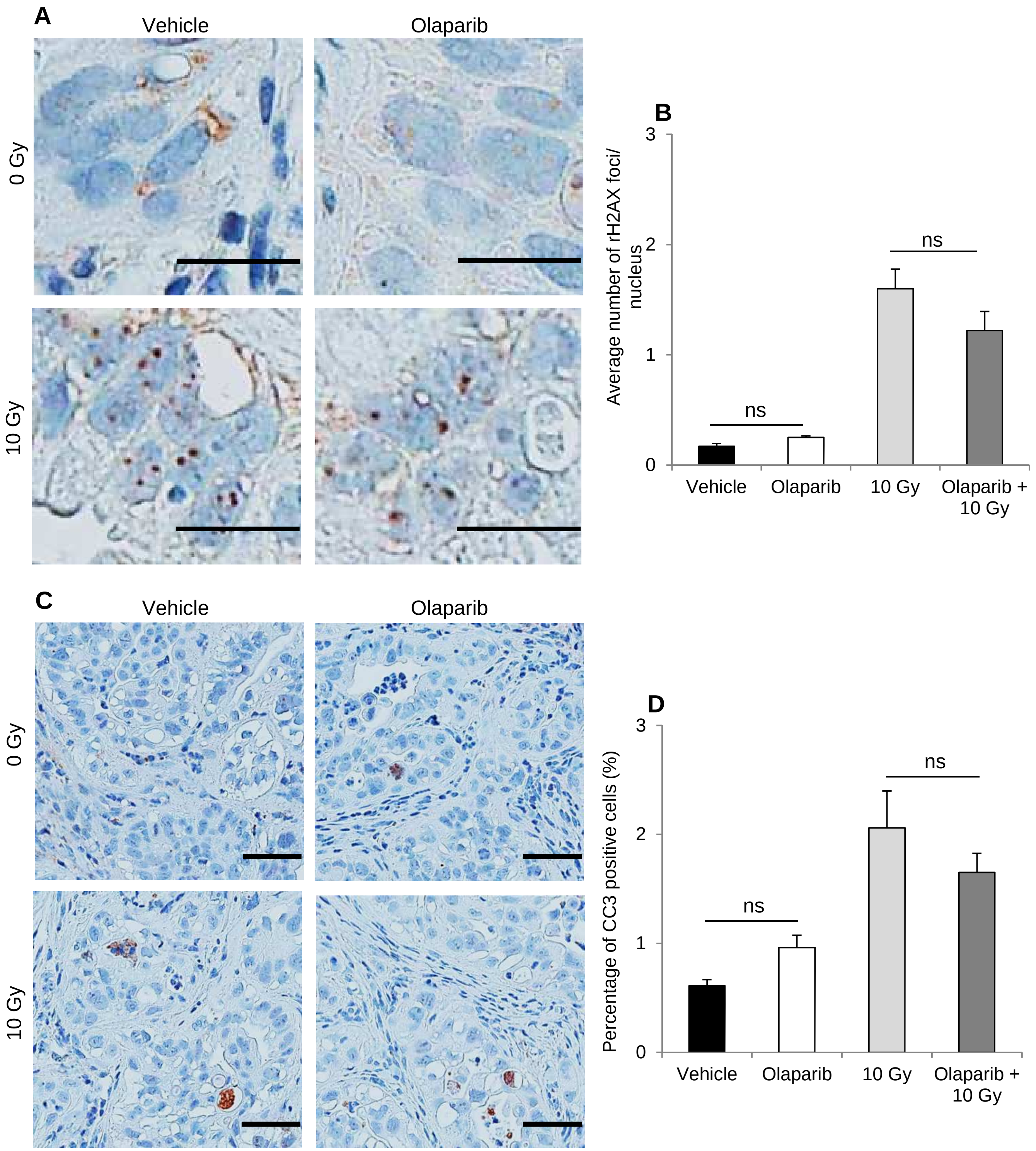

Supplementary Fig. S4 Olaparib does not affect radiation-induced DNA damage repair and apoptosis in Calu-3 xenografts. Mice bearing Calu-3 xenografts were treated with olaparib or vehicle 30 min prior to 0 Gy or 10 Gy radiation. Tumors were collected 24 h or 72 h post-radiation for γH2AX or CC3 IHC staining. (A) Representative γH2AX staining at 24 h time point. Scale bars, 20 μm. (B) Quantitative analysis of average γH2AX foci per nucleus in Calu-3 tumors (mean ± SEM). (C) Representative CC3 staining at 72 h time point. Scale bars, 50 μm. (D) Quantitative analysis of the percentage of CC3 positive cells in Calu-3 tumors (mean ± SEM). ns: not significant.

Supplementary Fig. S4
